# 基于随机扩散理论的气相色谱分离模拟

**DOI:** 10.3724/SP.J.1123.2021.10011

**Published:** 2022-03-08

**Authors:** Yinlu SUN, Lin WANG, Zhiyu YIN, Jianwei ZHAO

**Affiliations:** 1.辽宁大学化学院, 辽宁 沈阳 110036; 1. College of Chemistry, Liaoning University, Shenyang 110036, China; 2.嘉兴学院材料与纺织工程学院, 浙江 嘉兴 314001; 2. College of Materials and Textile Engineering, Jiaxing University, Jiaxing 314001, China

**Keywords:** 气相色谱, 分离, 扩散, 随机模拟, gas chromatography (GC), separation, diffusion, random simulation

## Abstract

色谱分离过程中的粒子扩散问题是色谱动力学研究的基础,深入理解粒子的扩散行为对优化分离操作条件、提升色谱性能和开发新型色谱柱尤为关键。现有的模拟方法多集中于局部过程的热力学研究,而整体的扩散分离过程报道并不多见。为此,该文基于微尺度受限空间内随机扩散的方法,通过动态追踪粒子的运动轨迹,实现粒子在气相色谱开管柱内的扩散全过程模拟。基于前期烷烃同系物的分离模拟研究,结合Kovats保留指数,分别建立了吸附步数与温度、吸附步数与碳数的函数关系,由此获得不同类型的同系物在不同温度条件下的分离参数系统。以醇类同系物的分离验证模拟的可靠性,结果表明保留时间的相对误差基本控制在5%以内,而峰宽相对误差在0.75%~60%之间。峰宽误差较大的原因在于:(1)参数化计算过程中未能充分迭代以及使用外推法;(2)模型中忽略了醇分子之间的氢键作用。该文提出的模拟方法虽然可以准确地预测色谱保留时间以及合理描述色谱峰的基本形貌特征,但尚有进一步发展空间,特别是增加对分子间相互作用的细节处理,例如可参考分子力学的方法建立分子间势函数和吸附步数的关系,利用分子力学计算的能量来取代参数化的吸附步数,从而实现更为精确的分离过程模拟。总体而言,该文所提出的模拟方法为优化色谱分离操作条件和开发新型色谱分离技术提供了有价值的参考。

## Abstract (en)

Understanding the diffusion behavior of particles during chromatographic analysis is critical for optimizing the operation conditions, improving the chromatographic performance, and designing a new separation device. Most of the existing simulations focus on chromatographic thermodynamics, while very few consider the overall diffusion and separation process. Herein, a new simulation method for gas chromatography separation was developed based on the random diffusion theory in microscale restricted space. This method retained the key information for controlling the diffusion behavior, simplified the interaction between the particles to be separated, and enlarged the time scale of each step one molecule walks. Thus, the operational efficiency could be significantly improved due to reduced calculation, and the entire diffusion process in the gas chromatography capillary column could be simulated. In this model, the capillary column was represented by a two-dimensional long and narrow rectangle where the particles to be separated randomly diffused. Besides, a directional velocity along the axis of the chromatographic column exerted on the particle represented the driving force of the mobile phase. If a particle collided with the inner wall of the column, it would remain at the collision position even after some time lapsed. When desorption occured, the particle would flow along with the mobile phase until its next adsorption on the surface. The interaction between the particle and the inner wall was expressed by the equivalent adsorption time. By dynamically tracking the trajectories of particles, the statistical distribution of time for the residence of the particles in the chromatographic column could be obtained, that is, the detection signal (retention time). Based on the previous simulation studies on the separation of *n*-alkane homologues, combined with the Kovats Retention Index, the functional relationships between adsorption steps and temperature as well as carbon number were established. As a result, the separation parameter systems for various homologues at different temperatures were set up. The separation of alcohol homologues at different temperatures was considered as an example to verify the reliability of the simulation method. The relative errors between the measured and simulated values were within 5% for the retention time and 0.75%-60% for the peak width. The reasons for the large relative errors in the peak width are summarized as follows. On the one hand, parameterization of alcohol homologues was performed on the basis of a previous study on the separation law of *n*-alkane. Given the limitations of the current computing capability, the insufficient iteration in the parameterized process led to large errors. In addition, the errors at different temperatures further accumulated in extrapolated approximations. On the other hand, the strong hydrogen bond force between the alcohol molecules was simplified with the elastic collision, which increased the magnitude of the errors. Although the simulation method proposed in this paper can accurately predict the retention time and reasonably describe the morphological characteristics of chromatographic peaks, there is still room for improvement. In particular, the description of the detailed interactions between molecules must be improved. For example, the method of molecular mechanics may be assistant with the investigation of the functional relationship between interaction potential and adsorption steps. The interaction potential calculated on the basis of molecular mechanics replaces the parameterized adsorption steps, yielding more accurate simulation results. In general, the simulation method proposed in this study is a valuable reference for the optimization of chromatographic operating conditions and for the development of new chromatographic techniques.

色谱法是分离分析中最为常见的方法之一,其分离过程中的粒子扩散问题是色谱动力学研究的基础。深入理解粒子在两相中的扩散和传质行为,从微观角度揭示待分离粒子扩散分离的本质,可有效控制峰展宽、提高分离度,为实现快速优化分离、提升色谱性能和改进色谱柱提供了重要的理论依据^[1,2]^。

关于粒子扩散问题的研究,实验上多采用荧光显微镜^[3,4,5]^和原子力显微镜^[6]^来追踪粒子的运动轨迹。这些技术有效提高了人们对微观运动的理解和认识。然而,基于当前技术水平的限制,仅依靠实验手段不足以获得较为全面的粒子扩散运动信息。随着计算机技术的快速发展,模拟方法逐渐成为分子运动研究的重要辅助手段。目前,关于描述色谱微观扩散的模拟方法主要包括分子动力学方法和蒙特卡洛方法。比如,针对高效液相色谱分离的分子动力学模拟研究中,Liang等^[7]^探究了四氢呋喃-甲醇-二氧化硅界面上芳香族化合物的分离机理;Hirano等^[8]^考察了精氨酸对中性、阴离子或阳离子树脂色谱中蛋白质洗脱的影响机制,证实了精氨酸作为洗脱剂可有效提高蛋白质的分离效率;还有一些学者研究手性固定相分子、溶剂分子和药物分子对映体之间的相互作用,以揭示对映体的分离机制^[9,10,11,12]^。Bishop等^[13]^利用蒙特卡洛模拟揭示了色谱峰形的不对称性将随待测物与固定相之间相互作用增强呈现非线性扩展趋势。Prochazka课题组长期致力于液相色谱的蒙特卡洛模拟研究,包括孔径效应对聚合物分离的影响^[14]^,聚合物在块状和多孔介质中的分配机制^[15]^,以及强吸附多孔介质中不同链结构聚合物的相平衡和构象行为^[16]^等。

目前分子动力学和蒙特卡洛这两种方法均用于处理色谱吸附热力学、待分离物质的性质以及溶剂化环境等局部问题,尚未用于色谱扩散分离的全过程模拟。这是因为色谱全过程模拟的计算量过大,模拟用时远远超过可接受的程度。为了既能保留扩散分离过程中的重要细节,同时又能兼顾全过程模拟,本文结合分子动力学和随机运动理论的优势提出了受限空间内随机扩散模拟方法。该方法具有以下特点:(1)放大了模拟的时间尺度,避免了分子动力学模拟所涉及的大量冗余信息,从而减小运算量,提高运算效率,使分子运动理论能够应用于大规模扩散过程模拟;(2)模型的可扩展性强,可进一步增加各种细节模型处理以符合实际研究需要;(3)应用范围广,该方法已被广泛应用于多种扩散和分离过程研究,包括膜渗透过程^[17,18]^和电化学过程^[19,20]^的粒子扩散行为模拟等,具有实用价值和现实意义。

针对色谱过程模拟,前期已根据粒子的基本运动规律,设计了二维受限空间内随机扩散仿真模型,并对粒子在气相色谱中的扩散行为进行了探索。例如,在填充柱模拟中,重点考查了固定相的填充率和排布方式、柱压和柱长对色谱动力学的影响^[21]^;在毛细管柱模拟中,引入吸附时间描述待分离粒子与固定相之间的相互作用,系统考察了各模拟参数对粒子的碰撞情况和峰宽的影响^[22]^,并实现了烷烃同系物在不同载气流速下的分离模拟^[23]^。基于上述研究基础,本文进一步建立了吸附步数与温度、吸附步数与碳数的函数关系,获得多种类型同系物在不同温度条件下的分离参数系统,并通过醇类同系物的分离模拟验证了该方法的可行性。

## 1 理论部分

通过建立随机扩散模型,实现粒子在色谱柱中的扩散全过程模拟,该模型具体包括色谱柱长度和内径、粒子扩散运动、粒子与色谱柱内壁间的相互作用3个部分的数学描述,然后建立了保留时间的计算方法。

### 1.1 色谱柱

如图1所示,采用一个二维狭长矩形代表气相色谱毛细管柱,矩形的长边和短边分别代表毛细管柱的长度和内径,分别为30 m和319.5 μm。

**图1:**
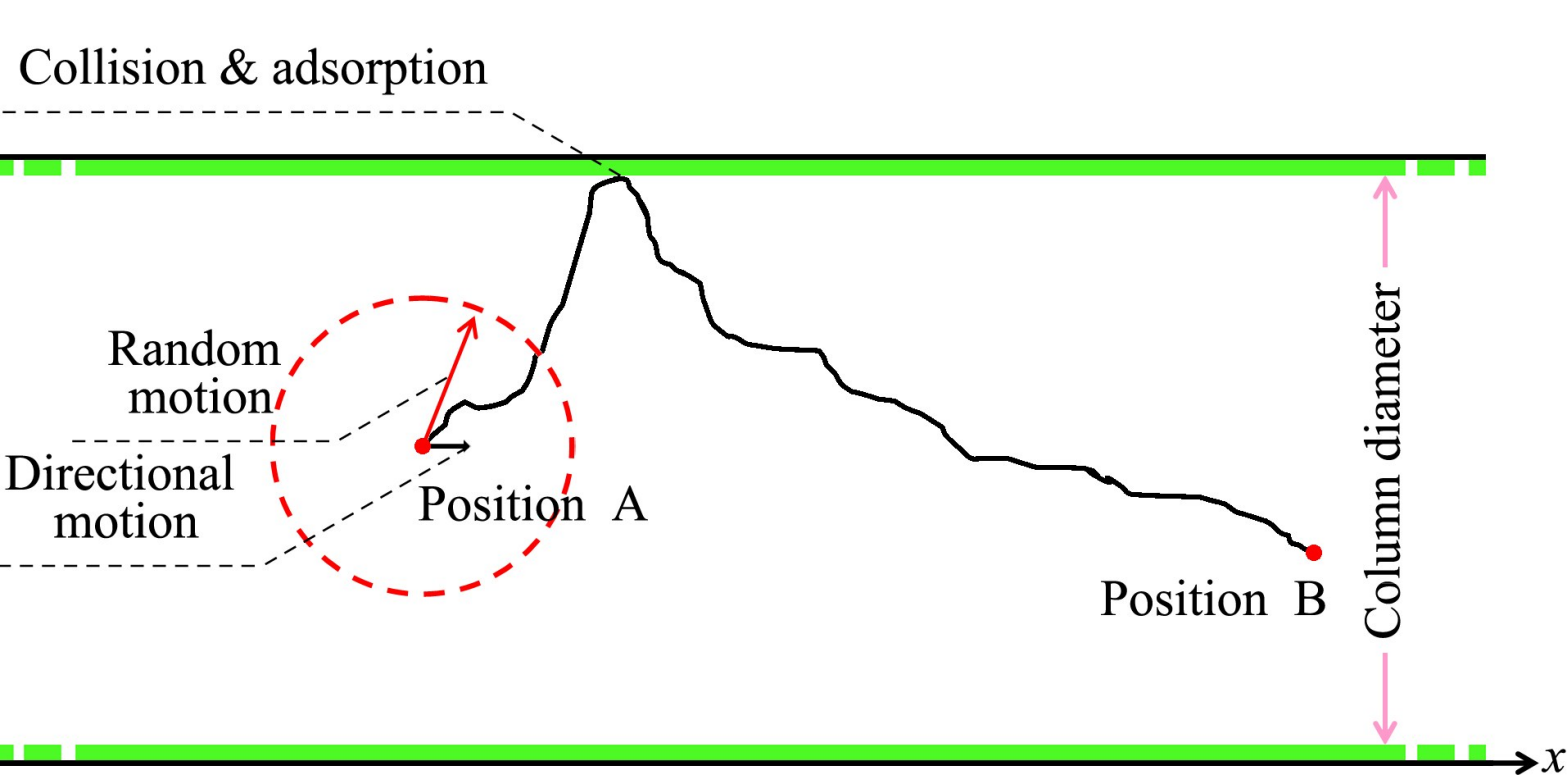
随机扩散模型示意图

### 1.2 粒子扩散运动的模型描述

采用质点表示待分离粒子,对待分离粒子质量和体积的考察可通过改变模拟条件等效实现。粒子在狭长矩形空间内部做随机运动,此随机运动被简化为全方向随机的匀速运动。粒子随机运动的每一步所用时间相同,记为时间步长,每一步的长度记为随机步长,随机速度为随机步长与时间步长的比值,即:


(1) $\delta=\frac{\lambda}{\Delta t}$

其中,*δ*为随机速度,*λ*为随机步长,Δ*t*为时间步长。

为了既保证扩散和分离关键细节模拟的精准度,又尽可能降低全过程模拟的高计算量,选取合适的随机步长和时间步长尤为关键。设置相对较大的随机步长和时间步长的优势在于,在把握粒子总体运动趋势的前提下,避免过多的无关分离过程的热运动描述,从而减少计算时间,提高计算效率。其中,随机步长是按照与毛细管柱内径成一定比例关系选取的,对于自由扩散,其比例系数为低于1/10(即32 μm)。时间步长确定了模拟的时间尺度,较小的步长有助于获得精确的运动轨迹,但计算量大、运算时间长。而随机扩散模型的特点就是在把握粒子总体运动趋势的前提下,尽可能将模拟的时间尺度放大,以避免热运动过程中的“冗余信息”。本文将时间步长设定为5×10^-6^s,该数值是经过多次模拟验证后确定的。随机速度实际上是一种表观速度(其值远低于粒子在真空中的热运动速度),它包含了与扩散相关的多种信息,不仅有粒子的热运动特征,还包括待分离粒子之间、待分离粒子和载气粒子之间、待分离粒子与毛细管柱壁之间的碰撞信息,是粒子热运动和多种相互作用的综合表现。

粒子除了随机运动外还需做定向运动。沿着色谱柱轴向对粒子施加一定向速度,用于描述载气对粒子的驱动作用。类似的,定向步长可用定向速度与时间步长的乘积计算得到,即:


(2) *l*=*u*_M_·Δ*t*

其中,*l*为定向步长,*u*_M_为定向速度。

### 1.3 相互作用模型

当粒子没有和毛细管柱壁碰撞时,粒子做随机扩散运动。根据随机速度、时间步长和粒子的当前位置即可确定粒子完成一步行走之后所处的位置。若粒子与毛细管柱壁发生碰撞时,引入吸附时间(即粒子停留在柱壁的平均时间)来描述粒子和毛细管柱壁之间的相互作用,当完成吸附行为后,粒子将会随着载气开始新的随机运动直到下一次吸附在毛细管柱壁表面。吸附时间可由吸附步数和时间步长的乘积求出,即:


(3) *t*=*n*_ads_·Δ*t*

其中,*t*为吸附时间,*n*_ads_为吸附步数。需要说明的是,这里的吸附步数只是为方便计算吸附时间,没有完整一步的概念,可为小数。

### 1.4 保留时间计算方法

粒子在固定相上的滞留时间,即调整保留时间,可由碰撞数和吸附时间的乘积求出:


(4) *t*_R_'=*N*_col_·*t*

其中,*t*_R_'为调整保留时间,*N*_col_为碰撞数。

而粒子在流动相中的滞留时间,即死时间,可由色谱柱长度和定向速度的比值求出:


(5) $t_{M}=\frac{L}{u_m}$

其中,*t*_M_为死时间,*L*为色谱柱长度。

结合式(3)、(4)和(5)得到粒子在色谱柱内的保留时间*t*_R_:


(6) *t*_R_=*t*_M_+*t*_R_'=$\frac{L}{u_m}$+*N*_col_·*n*_ads_·Δ*t*

由公式(6)可以看出,色谱的保留时间可分解成两个独立过程实现,其一是粒子在载气推动下的随机扩散运动,该过程在死时间内完成;其二是粒子与毛细管柱壁碰撞后的传质过程,该过程在调整保留时间内完成。应用上述单粒子扩散模型得到粒子的运动轨迹如图2a所示。在同一条件下实施多次模拟得到大量保留时间数据,对保留时间进行统计分析即可得到某一待测组分的色谱检测信号,见图2b。

**图2:**
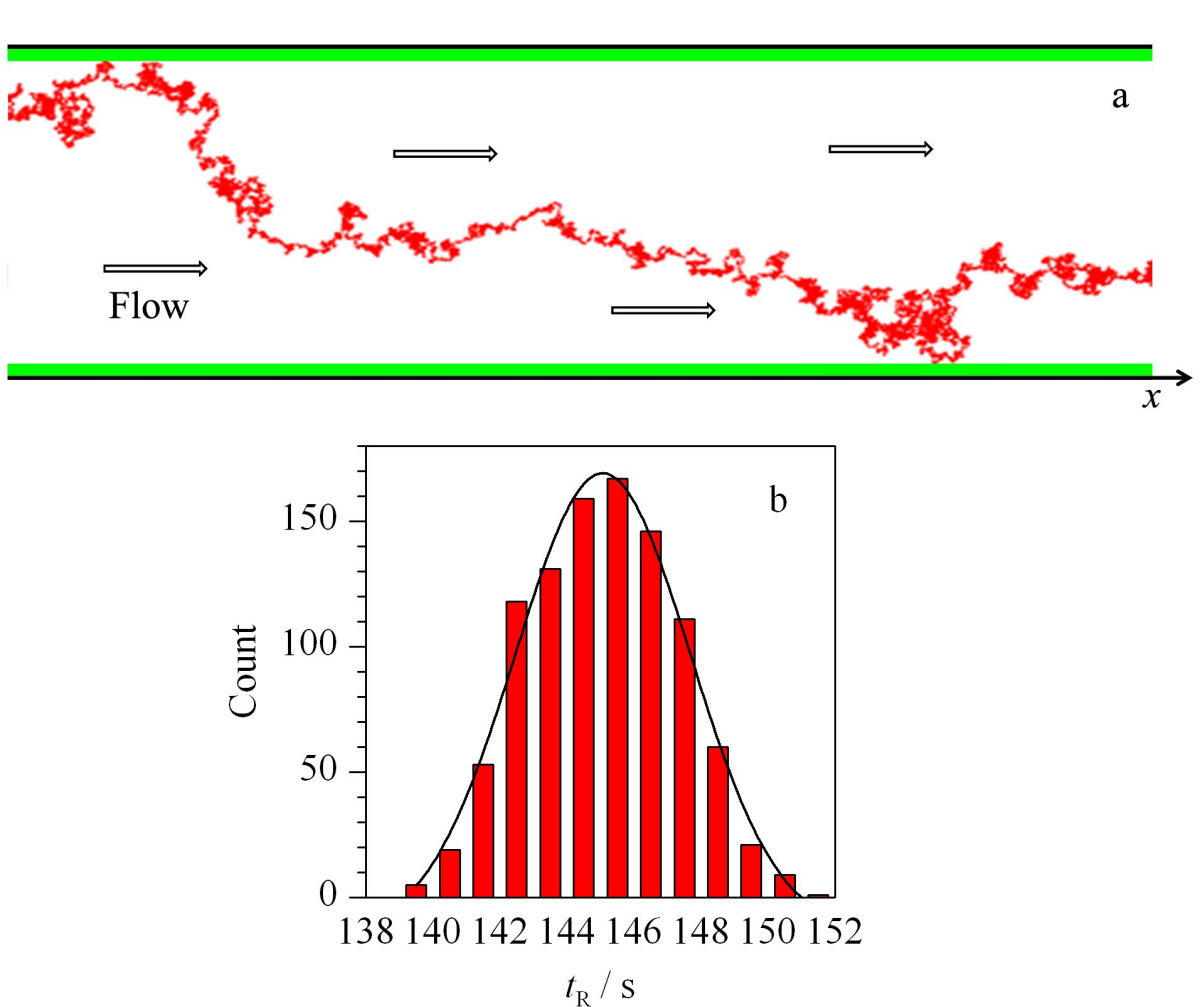
粒子的运动轨迹及其保留时间的统计分布示意图

由公式(6)可知,为了得到保留时间,需要明确柱长、粒子的定向速度、吸附步数、碰撞数和时间步长等模拟条件参数。根据我们之前的研究可知,柱长和粒子的定向速度与实验参数设定一致,随机速度和时间步长可由经验值给出,而碰撞数因为与随机速度和定向速度的比值呈线性关系也可预先估计^[23]^,因此吸附步数是模拟成功的关键因素。本文首先根据烷烃同系物的吸附步数探索其变化规律,再扩展到醇类同系物完成其吸附步数的参数化过程,最后实施醇类物质的分离模拟。

## 2 实验部分

实验采用配备氢火焰离子化检测器的气相色谱仪(安捷伦,7820A,中国)。毛细管柱采用HP-5弱极性柱(安捷伦,19091-413,中国),其长度为30 m,内径为319.5 μm。载气为高纯氮气(纯度为99.999%),流速为2.0 mL/min,尾吹流量为24 mL/min,氢气为30 mL/min,空气为300 mL/min,气化室温度和检测器温度均为483 K,柱温范围为333~393 K,分流比为1:80,进样量为0.2 μL,待测物为正辛烷(C_8_)、正壬烷(C_9_)、正癸烷(C_10_)、正十一烷(C_11_)和正十二烷(C_12_)的混合物。醇类待测物为正丙醇(C_3,OH_)、正丁醇(C_4,OH_)、正戊醇(C_5,OH_)、正己醇(C_6,OH_)、正庚醇(C_7,OH_)和正辛醇(C_8,OH_)的混合物,柱温为333 K和353 K,其他色谱条件同烷烃。所有试剂均从上海阿拉丁生化科技股份有限公司购买。

## 3 结果与讨论

### 3.1 吸附步数的变化规律

根据热运动理论,随机速度与柱温的平方根成正比,与组分相对分子质量的平方根成反比。若以正戊烷在333 K下的随机速度3.20 m/s为参考值,则其他正构烷烃在不同柱温下的随机速度可计算得出,结果见表1。定向速度与死时间成反比,对应实验设定的2.0 mL/min载气流速,不同温度下的定向速度参见表2。表1和表2数据均参考本课题组之前的研究方法^[23]^得到。

**表1:** 正构烷烃同系物在不同温度下的随机速度

T/K	δ/(m/s)				
	C_8_	C_9_	C_10_	C_11_	C_12_
348	2.60	2.45	2.33	2.22	2.13
353	2.62	2.47	2.35	2.24	2.14
358	2.64	2.49	2.36	2.25	2.16
363	2.66	2.51	2.38	2.27	2.17
368	2.67	2.52	2.40	2.29	2.19
373	2.69	2.54	2.41	2.30	2.20
378	2.71	2.56	2.43	2.32	2.22

**表2:** 粒子在不同温度下的定向速度

T/K	t_M_/min	u_M_/(m/s)	T/K	t_M_/min	u_M_/(m/s)
348	1.4038	0.3562	368	1.357	0.3685
353	1.3916	0.3593	373	1.3461	0.3714
358	1.3797	0.3624	378	1.3356	0.3744
363	1.3682	0.3655			

现已证明碰撞数与随机速度和定向速度的比值呈正相关^[23]^,即有:


(7) $N_{\mathrm{col}}=6.5 \times 10^{4} \frac{\delta}{u_{\mathrm{M}}}$

因此,若已知C_8_、C_9_、C_10_、C_11_和C_12_的保留时间实验值,即可根据公式(6)、(7)以及表1、表2的条件参数反推模拟所需的吸附步数。不同温度下测试烷烃同系物得到的保留时间实验值及其对应模拟所需的吸附步数列于表3中。

**表3:** 不同温度下的烷烃保留时间实验值及其对应的模拟所需吸附步数

T/K	t_Rexp_/min						n_ads_				
	C_8_	C_9_	C_10_	C_11_	C_12_		C_8_	C_9_	C_10_	C_11_	C_12_
348	2.36	3.36	5.41	9.59	18.14		24.29	52.64	113.41	243.11	519.06
353	2.19	3.01	4.66	8.01	14.77		20.34	43.60	92.89	196.91	415.74
358	2.07	2.72	4.00	6.49	11.35		17.55	36.27	74.53	152.39	310.34
363	1.96	2.50	3.53	5.48	9.22		15.11	30.56	61.41	122.82	244.64
368	1.87	2.31	3.13	4.67	7.52		13.09	25.84	50.68	98.92	192.29
373	1.79	2.16	2.83	4.04	6.25		11.45	22.08	42.32	80.73	153.37
378	1.73	2.03	2.58	3.54	5.24		10.07	18.94	35.44	65.96	122.26

根据上述吸附步数数据,总结吸附步数随色谱柱温度和待测分子所含碳原子数的变化规律。如图3a和3b所示,吸附步数的对数与温度的倒数呈线性关系,其斜率*a*为组分碳数的函数,表达式如下:


(8) $a=\frac{\ln n_{\mathrm{ads} 1}-\ln n_{\text {ads } 2}}{1000\left(\frac{1}{T_{1}}-\frac{1}{T_{2}}\right)}=0.64 n_{\mathrm{car}}-1.28$

**图3:**
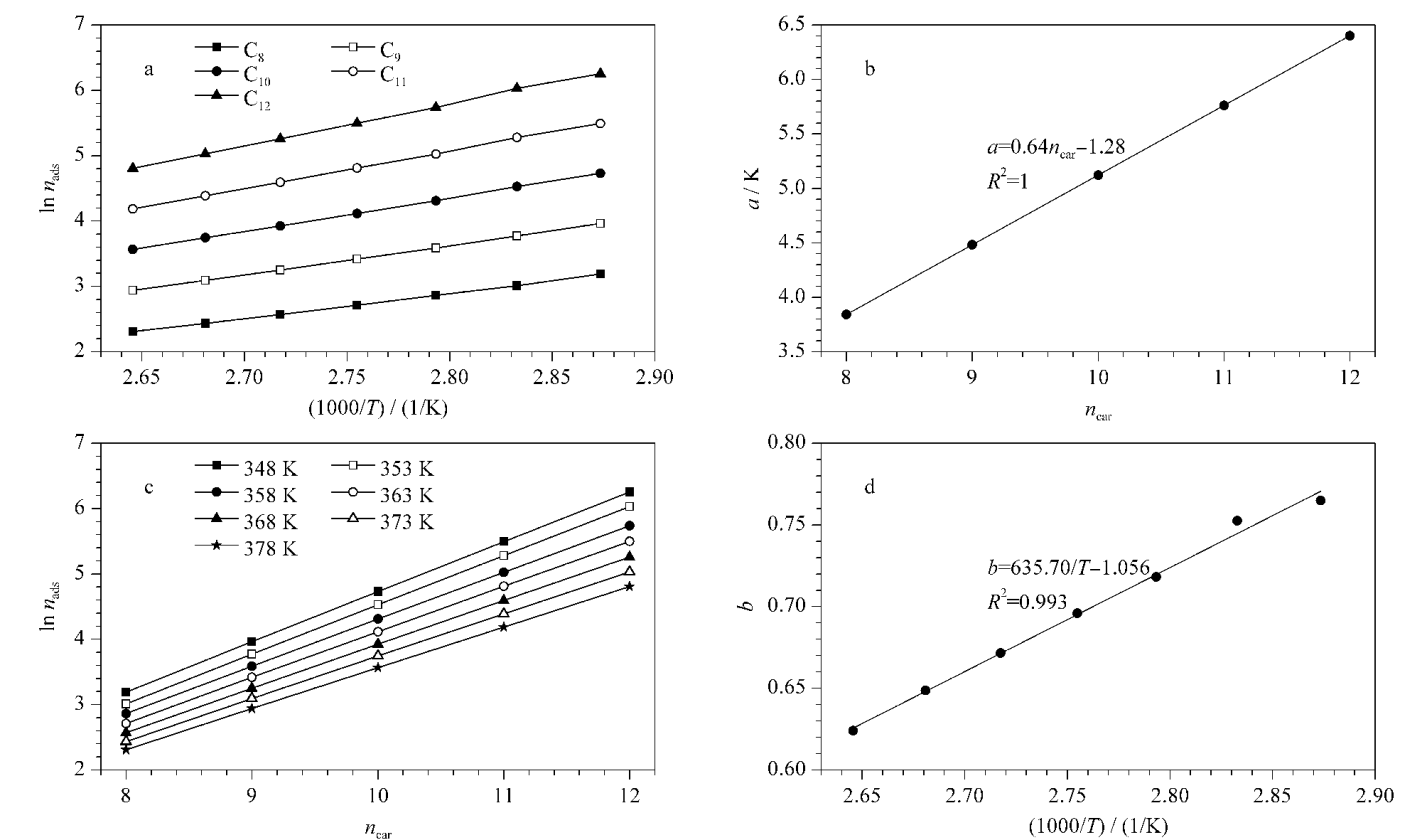
吸附步数随柱温、待测组分碳数的变化规律

其中640.19和1280.34是线性拟合参数,*n*_ads1_和*n*_ads2_分别对应待测组分在*T*_1_和*T*_2_温度下的吸附步数,*n*_car_为该组分分子所含碳原子数。由式(8)可知,若已知某组分在某一温度下的吸附步数,则可求出该组分在其他温度下的吸附步数。

如图3c和3d所示,在某一温度下吸附步数的对数和组分所含的碳原子数也存在线性关系,其斜率*b*被证明为温度倒数的函数,表达式如下:


(9) $b=\frac{\ln n_{\text {ads1 } 1}-\ln n_{\text {ads } 2}}{n_{\text {car1 }}-n_{\text {car2 }}}=635.70 \cdot \frac{1}{T}-1.056$

其中635.70和1.056是线性拟合参数,*n*_ads1_和*n*_ads2_分别对应组分1和组分2在某一温度下的吸附步数,*n*_car1_和*n*_car2_分别代表组分1和组分2的分子所含碳原子数。由式(9)可知,在某一温度下,若已知某组分的吸附步数则可计算出其他组分在该温度下的吸附步数。

### 3.2 醇类同系物的色谱分离预测

式(8)和(9)所描述的吸附步数与温度和组分碳数的线性关系只适用于正构烷烃同系物,对于其他类型同系物也会有类似的表达式,只是线性拟合参数不同。为了使烷烃的吸附步数规律应用于其他类物质,可引入Kovats保留指数。

Kovats保留指数规定在任何一种色谱条件下,对碳原子数为*n*的任何正构烷烃,其保留指数为100*n*^[24]^。待测物质的保留指数是与待测物质具有相同调整保留值的假想的正构烷烃碳原子数的100倍。通常以色谱图上位于待测物质两侧的相邻正构烷烃保留值为基准,用对数内插法求得其保留指数,公式如下^[24]^:


(10) $I=100 \cdot\left[\frac{\log X_{i}-\log X_{z}}{\log X_{(z+1)}-\log X_{z}}+z\right]$

其中,*I*为Kovats保留指数,*X*为调整保留时间或调整保留体积,*z*为在前面洗脱出来的正构烷烃的碳原子数,*z*+1为在待测物质之后出峰的正构烷烃的碳原子数。

由式(10)可知,保留指数是以正构烷烃系列为参比标准,表示一个组分的相对保留能力的大小。欲测定一个组分的保留指数,必须测定两个经过选择的相邻正构烷烃的调整保留值,而这两个调整保留值必须在被测组分的前后。对于醇类同系物的分离来说,若已知其中一个组分的保留指数,也就确定了该组分相当于几个碳原子数的正构烷烃分子,再根据式(8)和式(9)求算其他同系物在不同温度下的吸附步数。下面以1-OH醇类同系物C_3,OH_、C_4,OH_、C_5,OH_、C_6,OH_、C_7,OH_和C_8,OH_混合物在柱温为333 K和353 K时的分离为例验证上述模拟方法的可靠性。

根据热运动理论,若以正戊烷在333 K下的随机速度3.20 m/s为参考值,则可计算出醇类同系物在333 K和353 K时的随机速度,结果见表4,对应的定向速度分别为0.3465 m/s和0.3593 m/s。

**表4:** 醇类同系物在不同温度下的随机速度

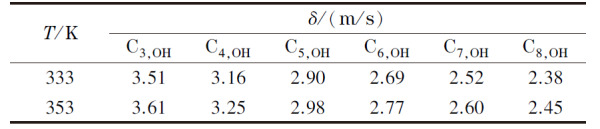

将333 K下正丁醇、正己烷、正庚烷的保留时间实验值代入公式(10)计算出正丁醇的保留指数为668,说明正丁醇在此色谱体系下的保留值相当于具有6.68个碳原子数的正构烷烃的保留值。已知正己烷在333 K下的吸附步数为7.36^[23]^,根据公式(9)可得出正丁醇的吸附步数为13.10,进而根据公式(8)和(9)确定醇类其他同系物在不同温度下的吸附步数,结果见表5。以表2、表4和表5中的模拟条件参数实施粒子的色谱分离过程模拟,对粒子走出色谱柱所需时间进行统计,通过高斯拟合得到色谱流出曲线,即色谱峰,结果表明,该模拟方法能够较为准确地预测各组分的保留时间及其色谱峰形的基本变化规律。将模拟的保留时间和峰宽(用标准偏差*σ*表示)与实验数据进行对比,其中,保留时间的模拟值*t*_Rsimu_、实验值*t*_Rexp_及其相对误差(Re)列于表6,峰宽的模拟值*σ*_simu_、实验值*σ*_exp_及其相对误差列于表7。

**表5:** 醇类同系物在不同温度下的吸附步数

T/K	Alkanol	Number of carbon atoms equivalent to n-alkanes	ln n_ads_	n_ads_
333	C_3,OH_	5.68	1.72	5.61
	C_4,OH_	6.68	2.57	13.10
	C_5,OH_	7.68	3.42	30.60
	C_6,OH_	8.68	4.27	71.46
	C_7,OH_	9.68	5.12	166.92
	C_8,OH_	10.68	5.97	389.86
353	C_3,OH_	5.68	1.32	3.76
	C_4,OH_	6.68	2.06	7.87
	C_5,OH_	7.68	2.80	16.48
	C_6,OH_	8.68	3.54	34.52
	C_7,OH_	9.68	4.28	72.31
	C_8,OH_	10.68	5.02	151.46

**表6:** 醇类同系物的保留时间模拟值和实验值

T/K	Alkanol	t_Rsimu_/min	t_Rexp_/min	Re/%
333	C_3,OH_	1.75	1.70	2.88
	C_4,OH_	2.09	2.01	3.86
	C_5,OH_	2.83	2.72	4.02
	C_6,OH_	4.44	4.31	3.08
	C_7,OH_	8.01	7.94	0.90
	C_8,OH_	15.92	16.13	1.33
353	C_3,OH_	1.60	1.57	1.65
	C_4,OH_	1.78	1.72	3.23
	C_5,OH_	2.13	2.04	4.46
	C_6,OH_	2.83	2.69	5.15
	C_7,OH_	4.21	4.04	4.22
	C_8,OH_	6.98	6.84	1.95

**表7:** 醇类同系物的峰宽模拟值和实验值

T/K	Alkanol	σ_simu_/min	σ_exp_/min	Re/%
333	C_3,OH_	0.225	0.453	-52.40
	C_4,OH_	0.234	0.475	-50.77
	C_5,OH_	0.449	0.630	-28.72
	C_6,OH_	0.996	1.004	-0.75
	C_7,OH_	2.196	2.067	6.24
	C_8,OH_	5.491	4.706	16.67
353	C_3,OH_	0.393	0.500	-61.42
	C_4,OH_	0.204	0.509	-59.97
	C_5,OH_	0.263	0.593	-55.60
	C_6,OH_	0.516	0.781	-33.94
	C_7,OH_	0.977	1.276	-23.40
	C_8,OH_	1.718	2.294	-25.13

本文提出了一种基于随机扩散理论的色谱模拟新方法,通过追踪1000个粒子在色谱柱中受驱动作用下的随机扩散轨迹,最后统计得出色谱的保留时间和峰宽值。对比实验数据可知,本文模拟的全部分子的保留时间其相对误差基本能控制在5%以内,表明该模拟方法可以准确地定性预测色谱分析结果以及描述色谱峰形的基本特征。即便从定量上考察峰宽,对于多数分子其相对误差也在0.75~50%之间,仅有个别体系(分子特别小,或者温度更高时)误差在60%左右。而应用同一方法,烷烃同系物其保留时间的相对误差可控制在3%以下,峰宽相对误差可控制在10%以下^[23]^。醇类物质比烷烃类物质误差增大的原因分析为以下两点:一方面,醇类物质的模拟是根据烷烃数据规律来实现参数化的,计算过程中未经充分迭代,而引入多次迭代则导致计算量过大,模拟时间过长,以现有的计算能力还不能完全实现。加之外推到不同温度下不同物质的分离模拟时,其误差还存在累积效应,从而进一步放大。另一方面,分离模型采用了弹性碰撞方式简化了醇分子间的相互作用,这种处理对于分子间作用力非常弱的烷烃类物质是准确的,而含有羟基的醇类物质之间有较强的分子间氢键作用,因而在模拟醇类同系物的分离时误差较大。本文提出的理论模型虽然可以准确地预测色谱保留时间以及合理预测色谱峰的形状和峰宽,但尚有进一步发展的空间。特别是增加对分子间相互作用的细节处理,以获得更加准确的模拟结果。例如可参考分子力学的方法,建立分子间势函数和吸附步数的关系。利用分子力学计算的能量来取代参数化的吸附步数,从而实现更为精确的分离过程模拟。此外,还可根据已知的相互作用推测分子结构碎片,分析待分离物质中可能存在的成分,为拓展分离分析提供更多的信息。

## 4 结论

本文基于微尺度受限空间内随机扩散理论,构建了气相色谱毛细管柱分离模型,实现了粒子在色谱柱内扩散分离的全过程模拟。在前期对烷烃同系物分离模拟研究的基础上,结合Kovats保留指数,分别建立了吸附步数与温度、吸附步数与分子碳数的函数关系,由此获得不同类型的同系物在不同温度条件下的分离参数系统。将该方法应用于醇类同系物在不同温度下的分离模拟,结果表明该方法能准确模拟色谱的保留时间和描述色谱峰的基本形貌特征。模拟的色谱峰宽误差较大,其原因可归结于参数化过程未充分迭代、外推法、醇分子之间氢键作用未计入三方面因素。在下一步研究中,有必要引入分子间相互作用力来代替现有的弹性碰撞模型,以获得更为准确的分离模拟结果。总体而言,本文所提出的模拟方法对优化色谱分离操作条件和开发新型色谱分离技术具有参考意义。
